# Supplementary material for: On a Non-Discrete Concept of Prokaryotic Species
Source: Microorganisms. 2020 Nov 4;8(11):1723. doi: 10.3390/microorganisms8111723 (PMC7692863; doi:10.3390/microorganisms8111723)
Supplement: Supplementary file 1 [file microorganisms-08-01723-s001.pdf]

Spplementary Material

On a non-discrete concept of prokaryotic species

Juan M. Gonzalez [1]*, Elena Puerta-Fernández [1], Margarida M. Santana [2] and Bhagwan Rekadwad [3]

[1] Instituto de Recursos Naturales y Agrobiología, Consejo Superior de Investigaciones Científicas, IRNAS-CSIC, Avda. Reina Mercedes 10, Sevilla, 41012, Spain
[2] Centre for Ecology, Evolution and Environmental Changes (cE3c), Faculdade de Ciências da Universidade de Lisboa, Edifício C2, Campo Grande, 1749-016 Lisboa, Portugal
[3] National Centre for Microbial Resource, National Centre for Cell Science, NCCS Complex, Savitribai Phule Pune University Campus, Ganeshkhind Road, Pune - 411007, Maharashtra State, India
* Correspondence: jmgrau@irnase.csic.es; Tel.: +34-95-462-4711

Journal: **Microoganisms**

Table S1. List of the example species analyzed in this study with the number of genomes available at NCBI (Microbial Genomes database) and the type strains used as reference.

Species	Number of strain genomes	Type strain
Bacillus subtilis	1116	NCIB 3610
Bacteroides fragilis	183	ATCC 25285
Clostridium botulinum	247	ATCC 25763
Helicobacter pylori	1677	ATCC 43504
Rhizobium leguminosarum	391	USDA 2370
Shigella boydii	112	ATCC 8700
Shigella flexneri	655	ATCC 24570
Shigella sonnei	1302	ATCC 29930
Streptococcus pyogenes	2130	CCUG 4207
Sulfolobus acidocaldarius	57	DSM 639
Vibrio cholerae	1441	ATCC 14035
